# Supplementary material for: Purification and biochemical analysis of native AMPA receptors from three different mammalian species
Source: PLoS One. 2023 Mar 17;18(3):e0275351. doi: 10.1371/journal.pone.0275351 (PMC10022779; doi:10.1371/journal.pone.0275351)
Supplement: S1 File — (PDF) [file pone.0275351.s001.pdf]

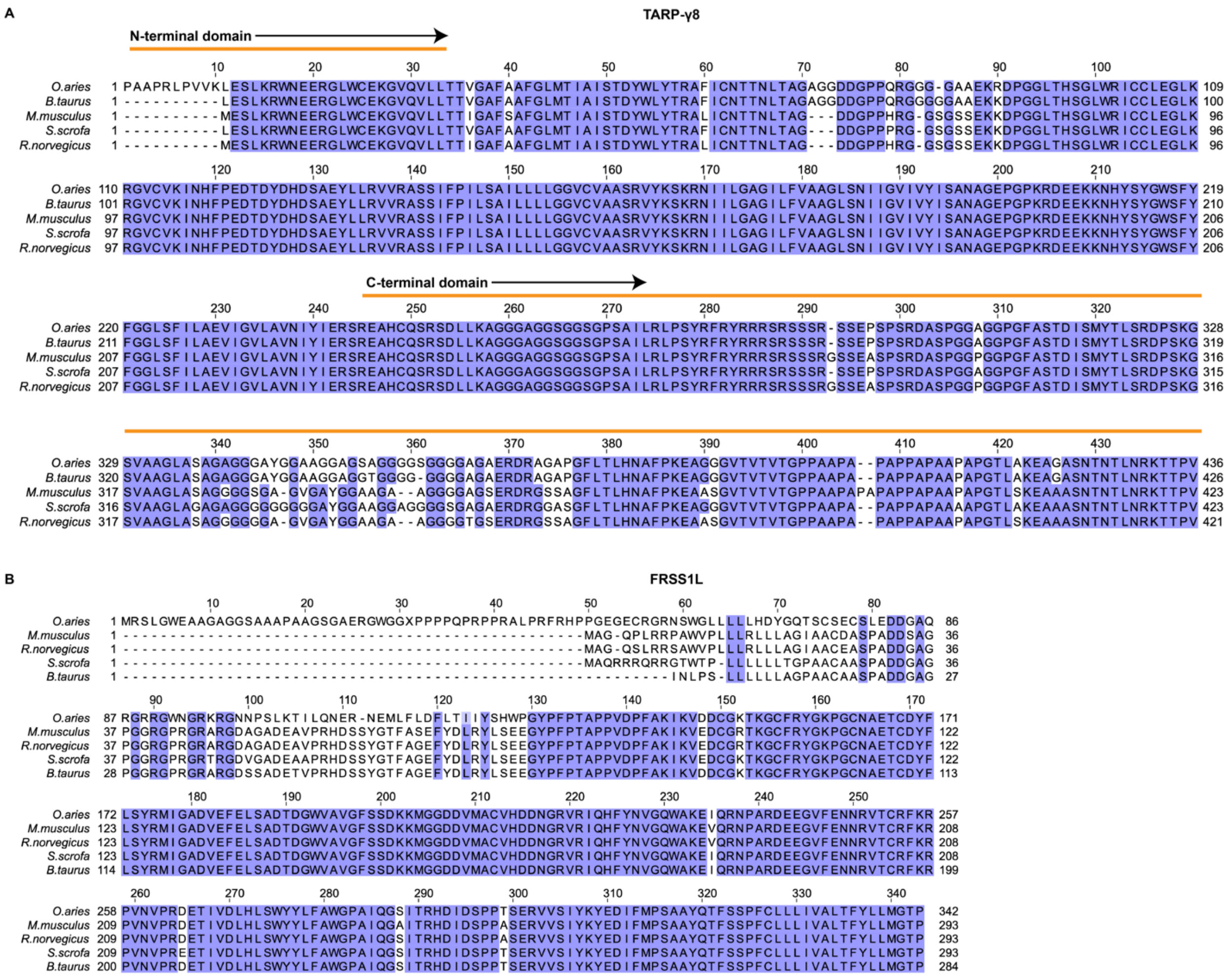


**S1 Figure. Sequence alignment of TARP-γ8 and FRRS1L**

(**A**) Sequence alignment of the five selected TARP-γ8 mammalian variants. Shaded regions highlight complete conservation across all species. (**B**) Sequence alignment of the five selected FRRS1L mammalian variants. Shaded regions highlight complete conservation across all species.

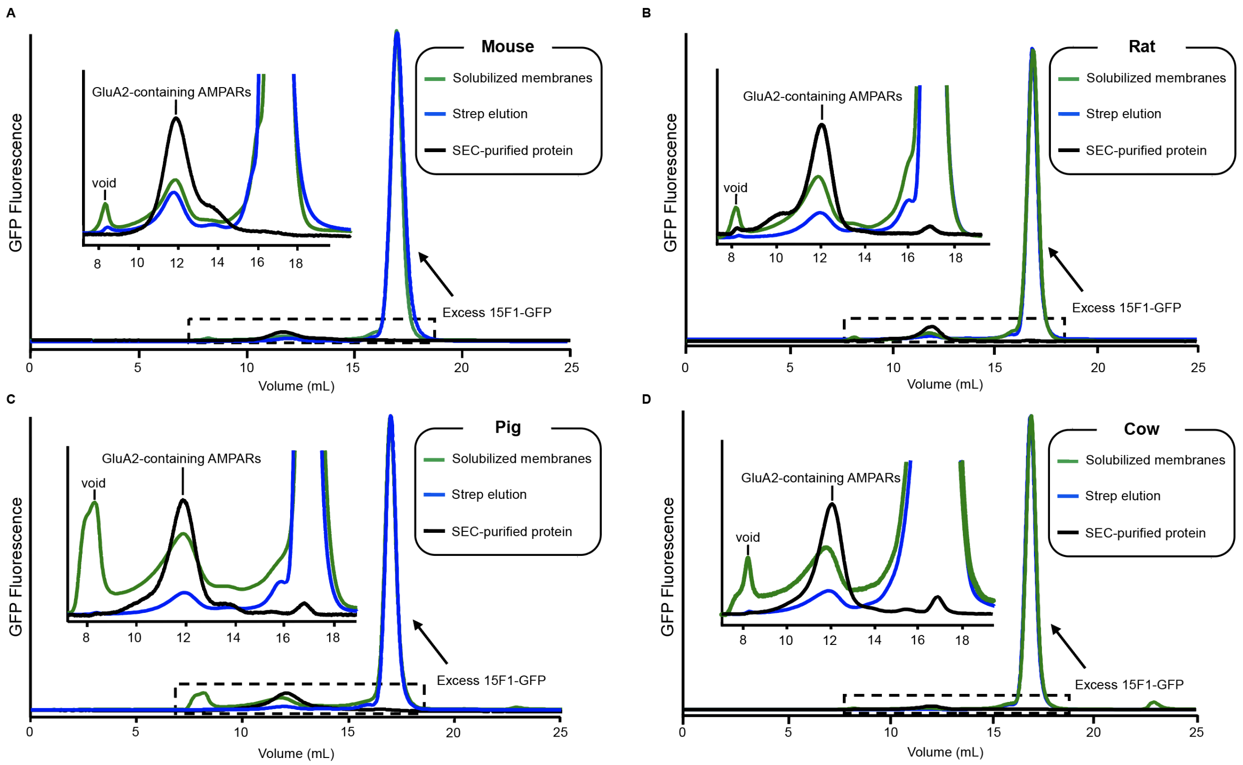


**S2 Figure. FSEC analysis of receptor complexes during immunoaffinity purification**

(**A-D**) Normalized FSEC profiles enable the visualization of the AMPAR complexes throughout the immunoaffinity purification workflow. Insets: Magnified views of the dashed rectangular regions, displaying peaks corresponding to native AMPARs.
