# Supplementary material for: Purification and biochemical analysis of native AMPA receptors from three different mammalian species
Source: PLoS One. 2023 Mar 17;18(3):e0275351. doi: 10.1371/journal.pone.0275351 (PMC10022779; doi:10.1371/journal.pone.0275351)
Supplement: S1 Raw images — (PDF) [file pone.0275351.s002.pdf]

Loading order:

1. sheep
2. cow
3. 15F1-GFP
4. pig
5. rat
6. mouse

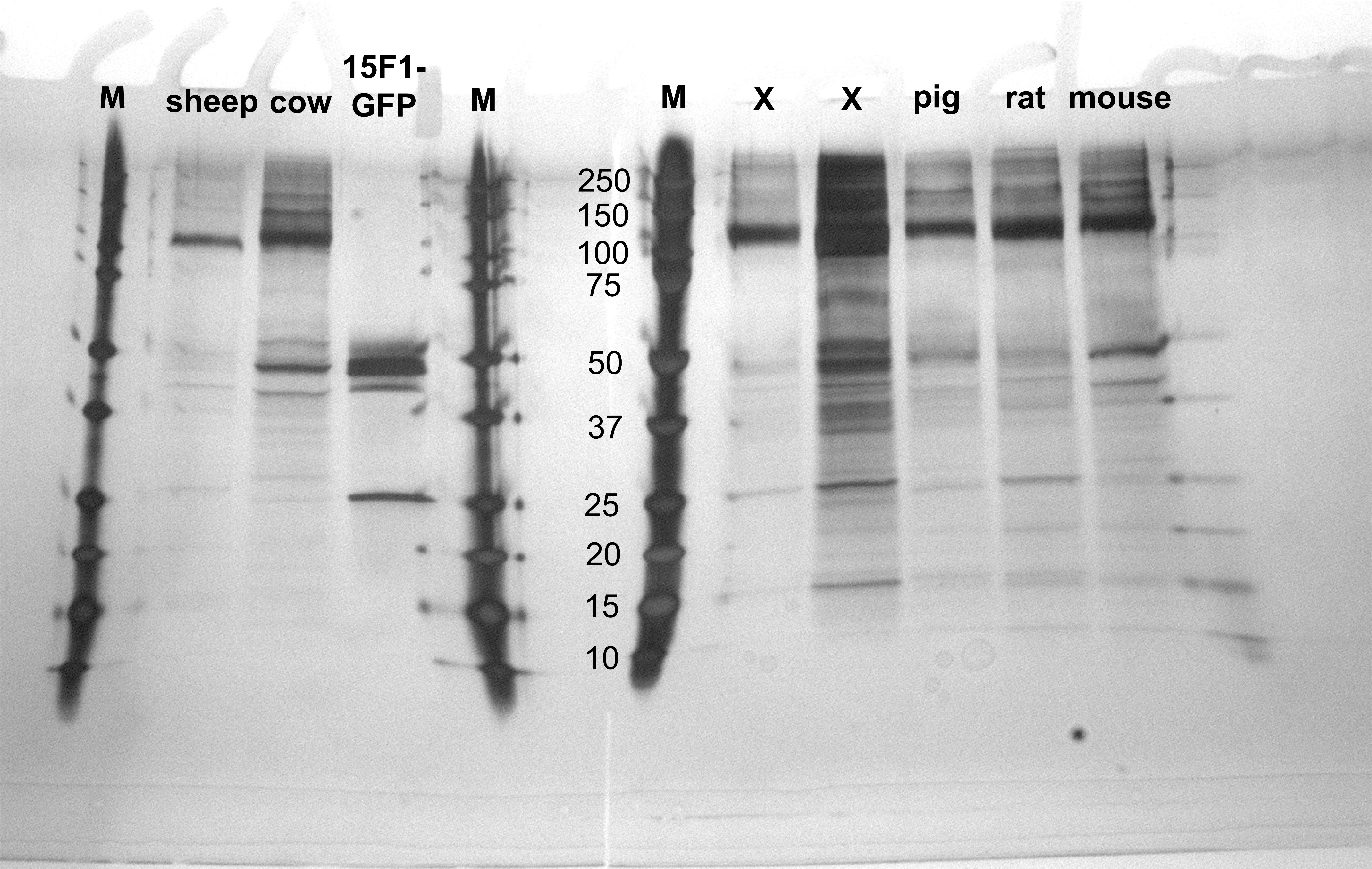

Gel image was captured using standard white light illumination settings

Original gels for Figure 4

Lanes labeled with “X” are excluded from Figure 4
